# MR 4D flow–derived left atrial acceleration factor for differentiating advanced left ventricular diastolic dysfunction

**DOI:** 10.1007/s00330-023-10386-9

**Published:** 2023-11-13

**Authors:** Clemens Reiter, Ursula Reiter, Corina Kräuter, Ewald Kolesnik, Daniel Scherr, Albrecht Schmidt, Michael Fuchsjäger, Gert Reiter

**Affiliations:** 1 Division of Neuroradiology, Vascular and Interventional Radiology, Department of Radiology, Medical University of Graz, Graz, Austria; 2 Division of Cardiology, Department of Internal Medicine, Medical University of Graz, Graz, Austria; 3 Division of General Radiology, Department of Radiology, Medical University of Graz, Auenbruggerplatz 9/P, 8036 Graz, Austria; 4 Research and Development, Siemens Healthcare Diagnostics GmbH, Graz, Austria

**Keywords:** Magnetic resonance imaging, Cardiovascular system, Diagnostic imaging, Heart function tests, Validation study

## Abstract

**Objectives:**

The magnetic resonance (MR) 4D flow imaging–derived left atrial (LA) acceleration factor *α* was recently introduced as a means to non-invasively estimate LA pressure. We aimed to investigate the association of *α* with the severity of left ventricular (LV) diastolic dysfunction using echocardiography as the reference method.

**Methods:**

Echocardiographic assessment of LV diastolic function and 3-T cardiac MR 4D flow imaging were prospectively performed in 94 subjects (44 male/50 female; mean age, 62 ± 12 years). LA early diastolic peak outflow velocity (*v*_*E*_), systolic peak inflow velocity (*v*_*S*_), and early diastolic peak inflow velocity (*v*_*D*_) were evaluated from 4D flow data. *α* was calculated from *α* = *v*_*E*_ / [(*v*_*S*_ + *v*_*D*_) / 2]. Mean parameter values were compared by *t*-test; diagnostic performance of *α* in predicting diastolic (dys)function was investigated by receiver operating characteristic curve analysis.

**Results:**

Mean *α* values were 1.17 ± 0.14, 1.20 ± 0.08, 1.33 ± 0.15, 1.77 ± 0.18, and 2.79 ± 0.69 for grade 0 (*n *= 51), indeterminate (*n *= 9), grade I (*n *= 13), grade II (*n *= 13), and grade III (*n *= 8) LV diastolic (dys)function, respectively. *α* differed between subjects with non-advanced (grade < II) and advanced (grade ≥ II) diastolic dysfunction (1.20 ± 0.15 vs. 2.16 ± 0.66, *p* < 0.001). The area under the curve (AUC) for detection of advanced diastolic dysfunction was 0.998 (95% CI: 0.958–1.000), yielding sensitivity of 100% (95% CI: 84–100%) and specificity of 99% (95% CI: 93–100%) at cut-off *α* ≥ 1.58. The AUC for differentiating grade III diastolic dysfunction was also 0.998 (95% CI: 0.976–1.000) at cut-off *α* ≥ 2.14.

**Conclusion:**

The 4D flow–derived LA acceleration factor *α* allows grade II and grade III diastolic dysfunction to be distinguished from non-advanced grades as well as from each other.

**Clinical relevance statement:**

As a single continuous parameter, the 4D flow–derived LA acceleration factor *α* shows potential to simplify the multi-parametric imaging algorithm for diagnosis of advanced LV diastolic dysfunction, thereby identifying patients at increased risk for cardiovascular events.

**Key Points:**

• *Detection of advanced diastolic dysfunction is typically performed using a complex, multi-parametric approach.*

• *The 4D flow–derived left atrial acceleration factor α alone allows accurate detection of advanced left ventricular diastolic dysfunction.*

• *As a single continuous parameter, the left atrial acceleration factor α could simplify the diagnosis of advanced diastolic dysfunction.*

## Introduction

Differentiation between left ventricular (LV) diastolic dysfunction with normal LV filling pressure from LV diastolic dysfunction with elevated LV filling pressure plays an important role in treatment and management of patients with heart failure. The LV filling pressure is considered elevated when the mean left atrial (LA) pressure, or its surrogate, the mean pulmonary arterial wedge pressure (PAWP), exceeds 15 mmHg [1]. Echocardiography represents the established non-invasive reference method for the evaluation and grading of LV diastolic dysfunction from a multi-parametric, threshold-based algorithm [2]. Cut-off values for the transmitral and mitral annular diastolic peak velocities and peak velocity ratios are employed together with cut-off values for the maximal tricuspid regurgitation velocity and the maximal LA volume to differentiate between normal LV diastolic function (grade 0 diastolic dysfunction), indeterminate diastolic function, and grades I–III diastolic dysfunction. Advanced LV diastolic dysfunction, defined as diastolic dysfunction of grade II or III, is considered to be associated with increased LV filling pressures [2]. The accurate detection of advanced diastolic dysfunction by echocardiography, however, can be challenging [3, 4].

The LA acceleration factor *α* derived from magnetic resonance (MR) 4D flow imaging was recently identified as a non-invasive means of estimating PAWP in subjects with pulmonary hypertension or at risk of having pulmonary hypertension [5]. Defined as the ratio of the 4D flow–derived early diastolic LA peak outflow velocity to the average of systolic and early diastolic LA peak inflow velocities, *α* correlated very highly with PAWP assessed by right heart catheterization and allowed the prediction of PAWP > 15 mmHg with high sensitivity and specificity.

As advanced LV diastolic dysfunction is expected to exhibit increased filling pressures [2], we hypothesized that *α*—as a single parameter—would allow discrimination between advanced and non-advanced forms of diastolic dysfunction. The aim of the present proof-of-principle study was therefore to investigate the relationship between *α* and the grades of LV diastolic dysfunction as evaluated from echocardiography.

## Materials and methods

### Study population

**Fig. 1:**
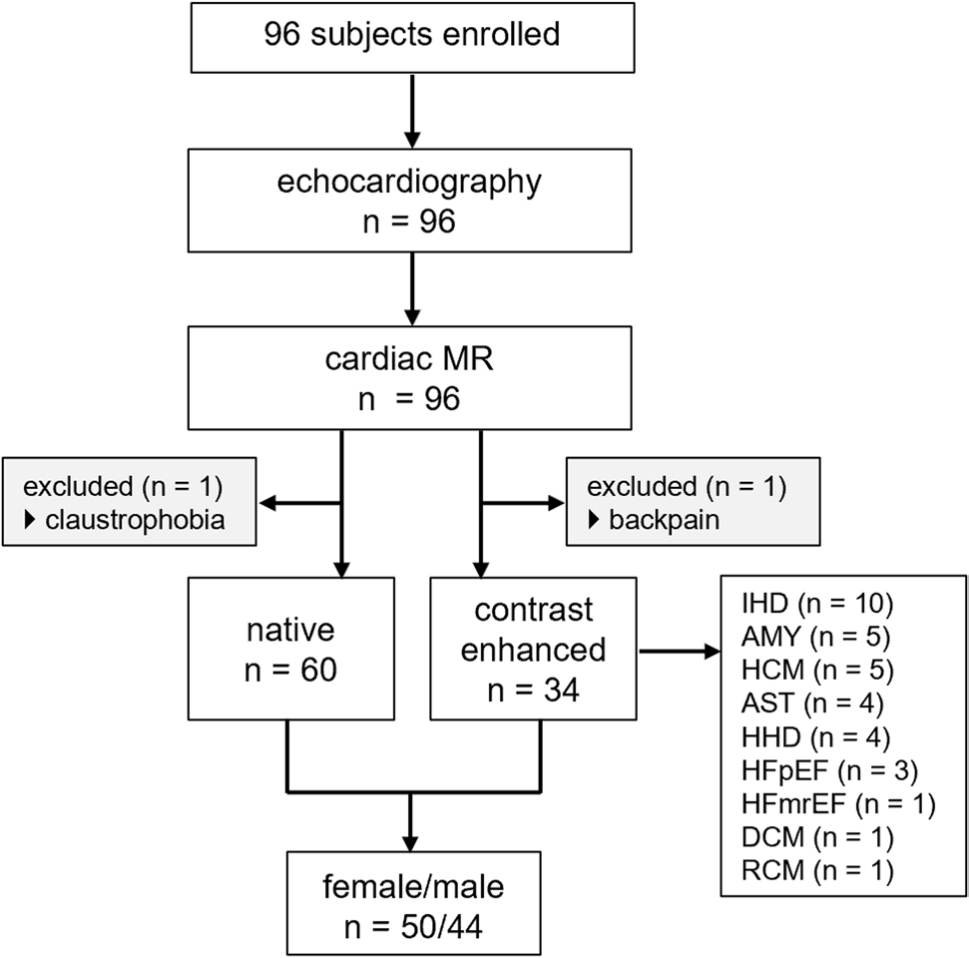
Study flowchart. Diagnosis for the patients undergoing the contrast enhanced examinations: IHD, ischemic heart disease; AMY, cardiac amyloidosis; HCM, hypertrophic cardiomyopathy; AST, aortic stenosis; HHD, hypertensive heart disease; HFpEF, heart failure with preserved ejection fraction; HFmrEF, heart failure with mildly reduced ejection fraction; DCM, dilatative cardiomyopathy; RCM, restrictive cardiomyopathy

Between October 2016 and February 2022, 96 adult subjects (age > 18 years) were prospectively recruited for echocardiography and MR 4D flow imaging. The study cohort consisted of 61 subjects without signs or symptoms of cardiovascular disease and without known cardiac disease (ClinicalTrials.gov, NCT01728597) and 35 consecutive subjects with referral for cardiac MR with known or suspected diastolic dysfunction documented in their medical records (ClinicalTrials.gov, NCT03253835). The study was approved by the local ethics review board and complied with the Declaration of Helsinki. Written informed consent was obtained from all participants. Exclusion criteria were contraindications for MR imaging, known pregnancy or claustrophobia, irregular heart rhythm, significant mitral stenosis, or implanted cardiac devices. Demographic (age, gender) and available clinical data (medical history, blood pressure, blood laboratory data) were collected at the time of inclusion. Two subjects did not complete cardiac MR imaging because of claustrophobia (*n* = 1) and severe back pain during the investigation (*n* = 1). Therefore, 94 patients were included in the analysis. Figure 1 presents the subject flowchart of the study.

### Transthoracic echocardiography and grading of diastolic (dys)function

Echocardiographic examinations were performed according to the 2016 American Society of Echocardiography (ASE) and European Association of Cardiovascular Imaging (EACVI) recommendations [2], using a Vivid E9 system (GE HealthCare). Briefly, images were acquired with simultaneous electrocardiographic recording during quiet respiration and with the patient in a left lateral decubitus position. The LV ejection fraction (EF) and maximal LA volume indexed to the body surface area (LAVI) were evaluated from apical 4-chamber and 2-chamber view series using the biplane Simpson method and the biplanar area-length method, respectively. Early diastolic and late diastolic transmitral peak velocities (referred to hereafter as *E* and *A*, respectively) were measured in the apical 4-chamber view. Early diastolic tissue Doppler mitral annular peak velocity *e*ʹ was calculated as the average of the septal and lateral velocities acquired in the apical 4-chamber view. The evaluation of the peak tricuspid regurgitation velocity (TR) was performed with continuous-wave Doppler in the apical 4-chamber view. Additionally, systolic and early diastolic pulmonary venous peak velocities (referred to hereafter as *S* and *D*, respectively) were measured in the right upper or right lower pulmonary vein, visualized in the apical 4-chamber view. After optimization of the transducer position, the Doppler sample volume was placed about 0.5 cm into the pulmonary vein. Moreover, the systolic pulmonary arterial pressure (sPAP) was estimated from TR (in m/s) via sPAP (in mmHg) = 4∙TR^2^ + 5 mmHg [6].

All echocardiographic parameters were evaluated from series of three to five consecutive cardiac cycles. Diastolic function was assessed employing the ASE/EACVI proposed algorithm [2]: In subjects with normal LV EF (≥ 50%), cut-offs for *e*ʹ, *E*/*e*ʹ, peak tricuspid regurgitation velocity, and LAVI were used to grade LV diastolic function as normal (grade 0, < 50% of echocardiographic cut-offs positive), indeterminate (50% positive), or diastolic dysfunction (> 50% positive). In subjects with diastolic dysfunction, as well as subjects with reduced LV EF or with a structural heart disease, grade of diastolic dysfunction (I–III) was determined using above parameter together with *E* and *E*/*A*.

### 4D flow imaging

MR 4D flow imaging was performed on a 3-T scanner (Magnetom Skyra, Siemens Healthineers) using a phased-array 18-channel body matrix together with a spine matrix coil. All subjects were investigated under shallow breathing in the supine position. Multislice 4D flow data were acquired from stacks of retrospectively electrocardiographically or pulse-gated 2-dimensional phase-contrast series with three-directional velocity encoding [7, 8], covering at least the LV and LA in 3-chamber-view orientation. In subjects referred for cardiac MR (*n* = 34), 4D flow imaging was performed 15–20 min after application of 0.2 mmol/kg gadobutrol (Gadovist; Bayer Schering Pharma). Parameters of the 4D flow protocol were as follows: voxel size, 1.8 × 2.5 × 4 mm^3^; measured temporal resolution, 41.8 ms interpolated to 30 cardiac phases per cardiac cycle; echo time, 3.1 ms; flip angle, 12° (native) or 15° (contrast enhanced); parallel acquisition factor, 2; velocity encoding, 100–190 cm/s in all directions (adjusted to prevent aliasing in LV and LA); number of averages, 2; typical acquisition time, 45 s per slice (50 heart beats) or 22 min to cover the heart (20–40 gapless slices).

### 4D flow data analysis

**Fig. 2:**
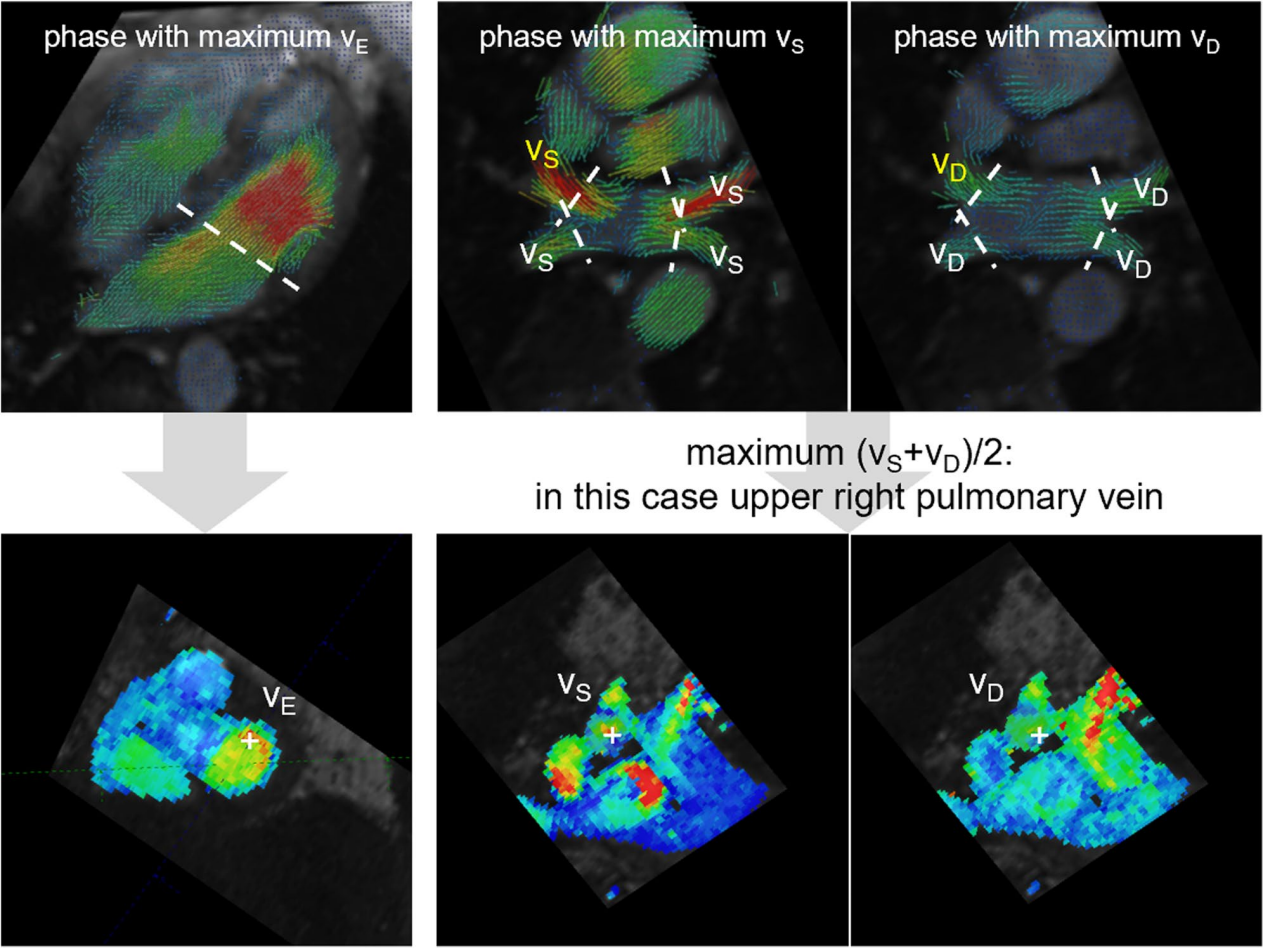
Evaluation of early diastolic LA peak outflow velocity (*v*_*E*_) at the atrioventricular junction (left) and LA peak inflow velocities during systole (*v*_*S*_) (middle) and early diastole (*v*_*D*_) (right) at the orifice of the pulmonary vein into the left atrium from 4D flow data. White dotted lines indicate the multiplanar reconstructed evaluation planes for peak velocities. Plus signs (lower panel) indicate the locations of the voxels with the peak velocities employed for calculation of the LA acceleration factor

Pre-processing (background phase correction, aliasing correction if necessary) and post-processing of 4D flow data were performed using prototype software (4Dflow, Siemens Healthineers). Early diastolic peak LA outflow velocity (*v*_*E*_) was assessed from the voxel with the highest velocity in an evaluation plane reconstructed parallel to the mitral valve at the level of the mitral valve annulus in early diastole. For the evaluation of the systolic (*v*_*S*_) and early diastolic (*v*_*D*_) peak LA inflow velocities, the cross sections of the left and right lower and upper pulmonary veins were reconstructed at the levels of the atrial junctions in systole and early diastole, respectively (Fig. 2). The typical duration for the total 4D flow analysis was 10 min per case. The evaluation was performed by a reader with 7 years of experience (C.R.). To investigate interobserver reliability of the derived variables, the evaluation was repeated for 20 randomly selected subjects by a second reader with 20 years of experience (U.R.), who was blinded to the previous results.

### LA acceleration factor

Following the definition of the LA acceleration factor *α* as the ratio of *v*_*E*_ to the average of *v*_*S*_ and *v*_*D*_ [5], *α* was calculated from 4D flow data according to the formula *α* = *v*_*E*_ / [(*v*_*S*_ + *v*_*D*_) / 2], where the maximum of *v*_*S*_ + *v*_*D*_ among all pulmonary veins was employed. Additionally, the LA acceleration factors *α*_lower_, derived by the same formula but using the maximum *v*_*S*_ + *v*_*D*_ among the lower pulmonary veins (as only these were evaluated in Reiter et al [5]), and *α*_echo_ = *E* / [(*S* + *D*) / 2], derived from corresponding echocardiographic Doppler measurements, were calculated for comparative purposes.

### Statistical analysis

Mean values are given together with standard deviations; areas under the curve (AUC), sensitivities, and specificities are specified together with 95% confidence intervals in parentheses. Statistical analysis was performed using SPSS^®^ v28 (IBM). For statistical tests, a significance level of 0.05 was employed.

Comparisons between corresponding parameters derived from MR and echocardiographic investigations or measurements employing all or only the lower pulmonary veins were performed by paired *t*-test after assessing normality by the Shapiro-Wilk test. Means of different groups of diastolic (dys)function were compared by analysis of variance with Dunett-T3 as a post hoc test; *α* for reduced and normal EF was compared by the Mann-Whitney *U* test. The relationships of continuous parameters were investigated by means of correlation, linear regression, and Bland-Altman analysis. Correlations are termed according to the correlation coefficients as negligible (0.0–0.3), low (0.3–0.5), moderate (0.5–0.7), high (0.7–0.9), or very high (0.9–1.0) [9]. Diagnostic performance of LA acceleration factors in discriminating advanced diastolic dysfunction was assessed with receiver operating characteristic (ROC) curve analysis, and 95% confidence intervals were calculated for area under the ROC curve (AUC), sensitivity, and specificity; non-overlapping confidence intervals were interpreted as different. Reported cut-off values correspond to the maximum of Youden’s index.

Interobserver reliabilities for determination of peak velocities and acceleration factors were determined by within-subject standard deviation (SD_w_) in variance component analysis and two-way mixed effect model, single measure, absolute agreement intra-class correlation coefficients (ICC). Interobserver reliability is termed poor (ICC 0.0–0.5), moderate (ICC 0.5–0.75), good (ICC 0.75–0.9), and excellent (0.9–1.0) [10].

## Results

### Study population

The analyzed study population consisted of 94 subjects (females/males, 50/44; age, 62 ± 12 years) who underwent echocardiography and cardiac MR imaging within 3 ± 7 days. The mean heart rates during echocardiography (68 ± 12 min^−1^) and 4D flow imaging (67 ± 11 min^−1^) did not differ (*p* = 0.334). Echocardiography diagnosed normal LV diastolic function in 51 subjects (54%), indeterminate LV diastolic (dys)function in 9 subjects (10%), and grade I diastolic dysfunction in 13 subjects (14%). Advanced LV diastolic dysfunction was diagnosed in 21 subjects (22%), with 13 subjects (14%) classified as having grade II and 8 subjects (9%) classified as having grade III diastolic dysfunction. The demographic characteristics of the study population are summarized in Table 1 together with echocardiographic and blood laboratory parameters. No subject had significant mitral or aortic regurgitation; 10 subjects demonstrated reduced EF.

**Table 1:** Baseline characteristics of the study population. Statistical test results for NTproBNP refer to the log-transformed quantity. Superscripts 0, i, 1, 2, and 3 indicate significant differences from grade 0, indeterminate, grade I, grade II, and grade III diastolic dysfunction, respectively. *BSA*, body surface area; *sBP*, systolic blood pressure; *dBP*, diastolic blood pressure; *HR*, heart rate; *eGFR*, estimated glomerular filtration rate; *NTproBNP*, N-terminal prohormone of brain natriuretic peptide; *HDL*, high-density lipoprotein; *LDL*, low-density lipoprotein; *EF*, ejection fraction; *E*, early diastolic transmitral peak velocity; *eʹ*, early diastolic mitral valve tissue peak velocity; *LAVI*, left atrial volume index; *TR*, peak tricuspid regurgitation velocity; *sPAP*, systolic pulmonary arterial pressure

Parameter	Total (*n *= 94)	Grade 0 (*n *= 51)	Indet. (*n *= 9)	Grade I (*n *= 13)	Grade II (*n *= 13)	Grade III (*n *= 8)	***p***
Demographic parameters							
Age (years)	62 ± 12	61 ± 9	59 ± 8	58 ± 17	68 ± 15	65 ± 14	0.212
Height (cm)	170 ± 10	171 ± 9	166 ± 8	172 ± 11	167 ± 10	171 ± 10	0.249
Weight (kg)	77 ± 15	75 ± 13	76 ± 21	80 ± 19	77 ± 13	86 ± 10	0.106
BSA (m^2^)	1.90 ± 0.21	1.88 ± 0.20	1.86 ± 0.27	1.95 ± 0.28	1.88 ± 0.18	2.01 ± 0.15	0.237
sBP (mmHg)	136 ± 19	134 ± 17	144 ± 15	138 ± 20	140 ± 26	136 ± 20	0.578
dBP (mmHg)	76 ± 11	75 ± 10	77 ± 7	73 ± 9	77 ± 15	86 ± 12	0.790
HR (min^−1^)	67 ± 11	67 ± 10	66 ± 9	69 ± 10	61 ± 11	75 ± 17	0.079
Laboratory parameters							
Hb (g/L)	14 ± 2	14 ± 1	14 ± 1	15 ± 2	14 ± 1	13 ± 3	0.065
eGFR (mL/min)	76 ± 18	80 ± 10^3^	82 ± 13^3^	78 ± 25^3^	74 ± 22	48 ± 16^i,1,2^	< 0.001
NTproBNP (pg/mL)	1298 ± 5986	76 ± 124^2,3^	84 ± 53^2,3^	561 ± 647^3^	689 ± 770^0,i,3^	15233 ± 18651^0,i,1,2^	< 0.001
Triglycerides (mg/dL)	113 ± 60	105 ± 49	97 ± 63	141 ± 49	132 ± 96	114 ± 76	0.075
HDL (mg/dL)	62 ± 20	65 ± 21	72 ± 23	50 ± 16	57 ± 12	48 ± 13	0.029
LDL (mg/dL)	135 ± 43	154 ± 31^1,2^	136 ± 45	99 ± 34^0^	92 ± 40^0^	121 ± 44	< 0.001
Echocardiographic parameters							
EF (%)	59 ± 10	61 ± 6	61 ± 5	60 ± 11	55 ± 14	52 ± 19	.068
*E* (cm/s)	82 ± 21	78 ± 16^3^	79 ± 15^3^	74 ± 13^3^	92 ± 32	109 ± 18^0,i,1^	< 0.001
*E*/*A*	1.23 ± 0.54	1.10 ± 0.29^3^	1.14 ± 0.27^3^	1.03 ± 0.32^3^	1.16 ± 0.35^3^	2.66 ± 0.53^0,i,1,2^	< 0.001
*e*ʹ (cm/s)	8.5 ± 7.1	10.6 ± 9.0^2,3^	7.9 ± 1.4^2,3^	6.6 ± 2.6	5.4 ± 1.3^0,i^	4.1 ± 1.8^0,i^	0.023
*E*/*e*ʹ	12.7 ± 8.8	8.3 ± 2.2^i,2,3^	10.0 ± 1.1^0,2,3^	13.2 ± 6.2	19.6 ± 5.7^0,i^	31.8 ± 14.3^0,1^	< 0.001
LAVI (mL/m^2^)	34 ± 13	28 ± 6^i,2^	36 ± 4^0^	32 ± 11^2^	51 ± 18^0,1^	46 ± 17	< 0.001
TR (cm/s)	2.4 ± 0.4	2.2 ± 0.1^2^	2.3 ± 0.3	2.1 ± 0.5	2.7 ± 0.4^0^	2.9 ± 0.5	< 0.001
sPAP (mmHg)	27.9 ± 8.1	25.0 ± 2.6^2^	25.6 ± 5.2	23.7 ± 7.7^2^	35.1 ± 8.8^0,1^	40.7 ± 11.4	< 0.001

### LA acceleration factor *α*

**Fig. 3:**
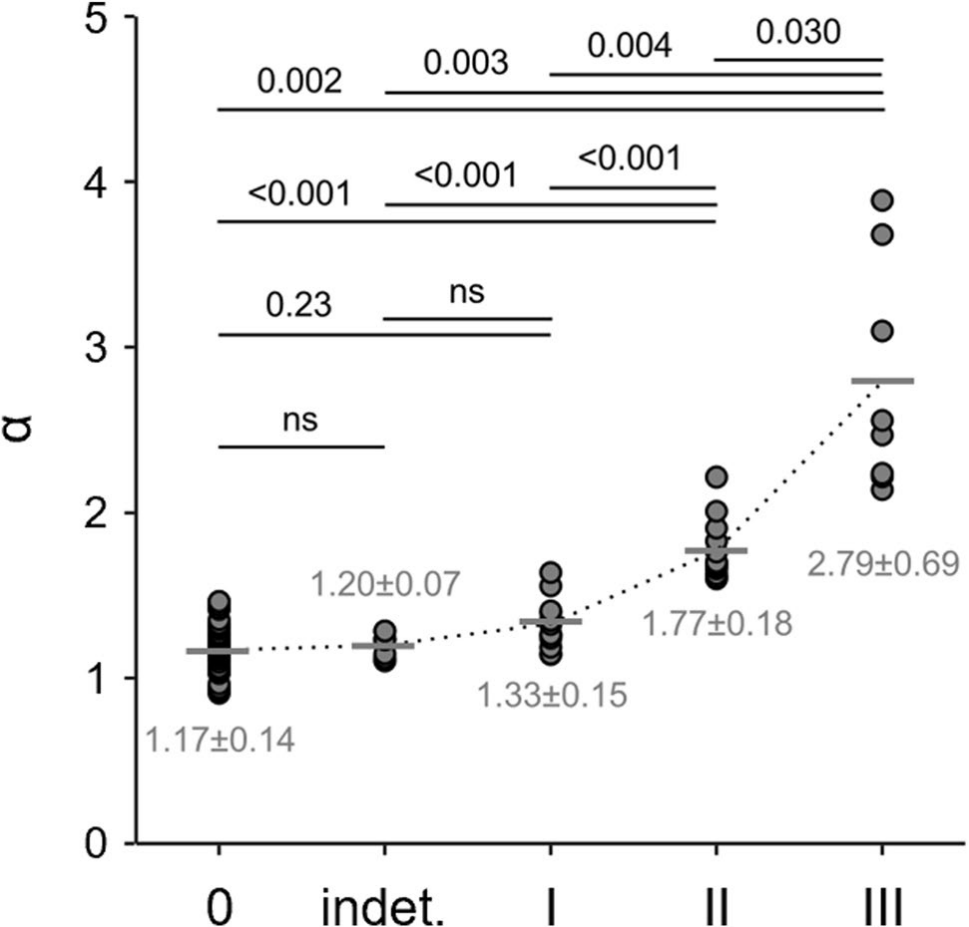
Dot-plot showing the LA acceleration factor for different groups of LV diastolic dysfunction. Comparisons of group means by the Dunett-T3 post hoc test are indicated by lines together with *p*-values. ns, non-significant; indet., indeterminate

Means of peak LA inflow and outflow velocities as well as corresponding LA acceleration factors for the total study population and for the groups of patients with diastolic dysfunction are summarized in Table 2. In 83 subjects (88%), the highest average LA inflow velocities were measured in the upper left (56%) or upper right (32%) pulmonary vein; the highest average LA inflow velocities were observed in the left lower pulmonary vein in 8 subjects (9%) and in the right lower pulmonary vein in 3 subjects (3%). *α* correlated significantly with all continuous echocardiographic parameters used for grading: EF (*r* = −0.27, *p* = 0.01), *E* (*r* = 0.40, *p* < 0.001), *E*/*A* (*r* = 0.60, *p* < 0.001), *e*ʹ (*r* = −0.37, *p* = 0.009), *E*/*e*ʹ (*r* = 0.66, *p* < 0.001), LAVI (*r* = 0.43, *p* < 0.001), TR (0.42, *p* < 0.001), and sPAP (*r* = −0.494, *p* < 0.001). *α* differed between subjects with normal and reduced EF (1.35 ± 0.46 vs. 1.99 ± 0.70, *p *< 0.001). Moreover, there were significant correlations of *α* with laboratory parameters Hb (*r* = −0.25, *p* = 0.02), eGFR (*r* = −0.49, *p* < 0.001), NTproBNP (*r* = 0.53, *p* < 0.001), and LDL (*r* = −0.34, *p* < 0.001). Maximum *v*_*E*_, *v*_*S*_, (*v*_*S*_ + *v*_*D*_) / 2, and *α* differed among the groups of patients with diastolic (dys)function, whereby means of *v*_*S*_ and (*v*_*S*_ + *v*_*D*_) / 2 decreased while *α* increased with grade of diastolic dysfunction. Notably, means of *α* for diastolic dysfunction grade II and grade III differed from those of all other groups (Fig. 3).

**Table 2:** LA peak inflow and outflow velocities and left atrial acceleration factor determined among all or only the lower pulmonary veins using MR 4D flow imaging data. Superscripts 0, i, 1, 2, and 3 indicate significant differences from grade 0, indeterminate, grade I, grade II, and grade III diastolic dysfunction, respectively. *v*_*E*_, early diastolic left atrial peak outflow velocity; *v*_*S*_, systolic left atrial peak inflow velocity; *v*_*D*_, early diastolic left atrial peak inflow velocity; *α*, left atrial acceleration factor from all pulmonary veins; *α*_*lower*_, left atrial acceleration factor derived from only the lower pulmonary veins

Parameter	Total (*n *= 94)	Grade 0 (*n *= 51)	Indet. (*n *= 9)	Grade I (*n *= 13)	Grade II (*n *= 13)	Grade III (*n *= 8)	*p*
All pulmonary veins							
*v*_*E*_ (cm/s)	57.0 ± 13.2	52.8 ± 8.3^2,3^	51.5 ± 6.7^3^	57.0 ± 10.8	61.1 ± 7.8^0^	83.7 ± 21.7^0,i^	< 0.001
Maximum *v*_*S*_ (cm/s)	50.1 ± 11.7	54.4 ± 9.0^2,3^	52.1 ± 8.7^3^	50.5 ± 10.1^3^	43.0 ± 10.9^0^	30.9 ± 11.2^0,i,1^	< 0.001
Maximum *v*_*D*_ (cm/s)	36.2 ± 9.4	38.1 ± 8.5	35.8 ± 4.4	37.1 ± 11.4	29.8 ± 8.4	34.0 ± 13.7	0.072
Maximum (*v*_*S*_ + *v*_*D*_) / 2 cm/s	43.1 ± 9.1	46.2 ± 7.6^2^	43.9 ± 6.1	43.8 ± 8.8	36.4 ± 7.2^0^	32.5 ± 11.5	< 0.001
*α*	1.41 ± 0.50	1.17 ± 0.14^1,2,3^	1.20 ± 0.07^2,3^	1.33 ± 0.15^0,2,3^	1.77 ± 0.18^0,i,1,3^	2.79 ± 0.69^0,i,1,2^	< 0.001
Lower pulmonary veins							
Maximum (*v*_*S*_ + *v*_*D*_) / 2 cm/s	35.6 ± 8.4	37.0 ± 7.4	38.8 ± 5.4	36.9 ± 11.0	31.1 ± 6.3	28.6 ± 10.6	0.012
*α*_lower_	1.68 ± 0.59	1.46 ± 0.23^2,3^	1.33 ± 0.12^1,2,3^	1.60 ± 0.24^i,2,3^	2.00 ± 0.30^0,i,1^	3.14 ± 0.87^0,i,1^	< 0.001

**Fig. 4:**
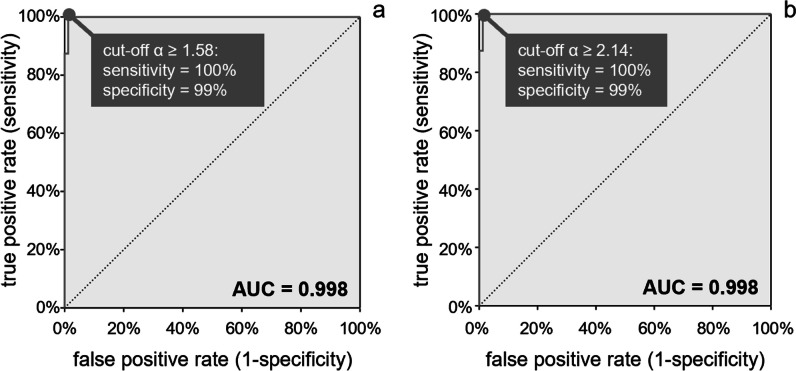
Receiver operator characteristic curve analysis for prediction of (**a**) advanced diastolic dysfunction (grade ≥ II) and (**b**) grade III diastolic dysfunction. Cut-off values at maximum of Youden’s index are given together with their sensitivity and specificity. AUC, area under the curve

Compared to normal, indeterminate, and grade I diastolic dysfunction, *α* was higher in advanced diastolic dysfunction (1.20 ± 0.15 vs. 2.16 ± 0.66, *p* < 0.001). The AUC for the detection of advanced diastolic dysfunction from *α* was 0.998 (0.958–1.000) (Fig. 4). With the cut-off value *α* ≥ 1.58, sensitivity and specificity for detection of advanced diastolic dysfunction were 100% (84–100%) and 99% (93–100%), respectively. The AUC for differentiation of grade III diastolic dysfunction was 0.998 (0.976–1.000). Using a cut-off value *α* ≥ 2.14, sensitivity and specificity for detection of grade III diastolic dysfunction were 100% (63–100%) and 99% (94–100%), respectively.

Within the subjects with non-advanced diastolic dysfunction, the AUC for detection of normal diastolic function from *α* was 0.711 (0.569–0.812) (sensitivity and specificity of 71% (56–83%) and 68% (45–86%) at a cut-off value *α* ≤ 1.22) and 0.797 (0.646–0.888) for detection of grade I diastolic dysfunction (sensitivity and specificity of 77% (46–95%) and 72% (59–83%) at a cut-off value *α* ≥ 1.24).

### LA acceleration factor from lower pulmonary veins *α*_lower_

**Fig. 5:**
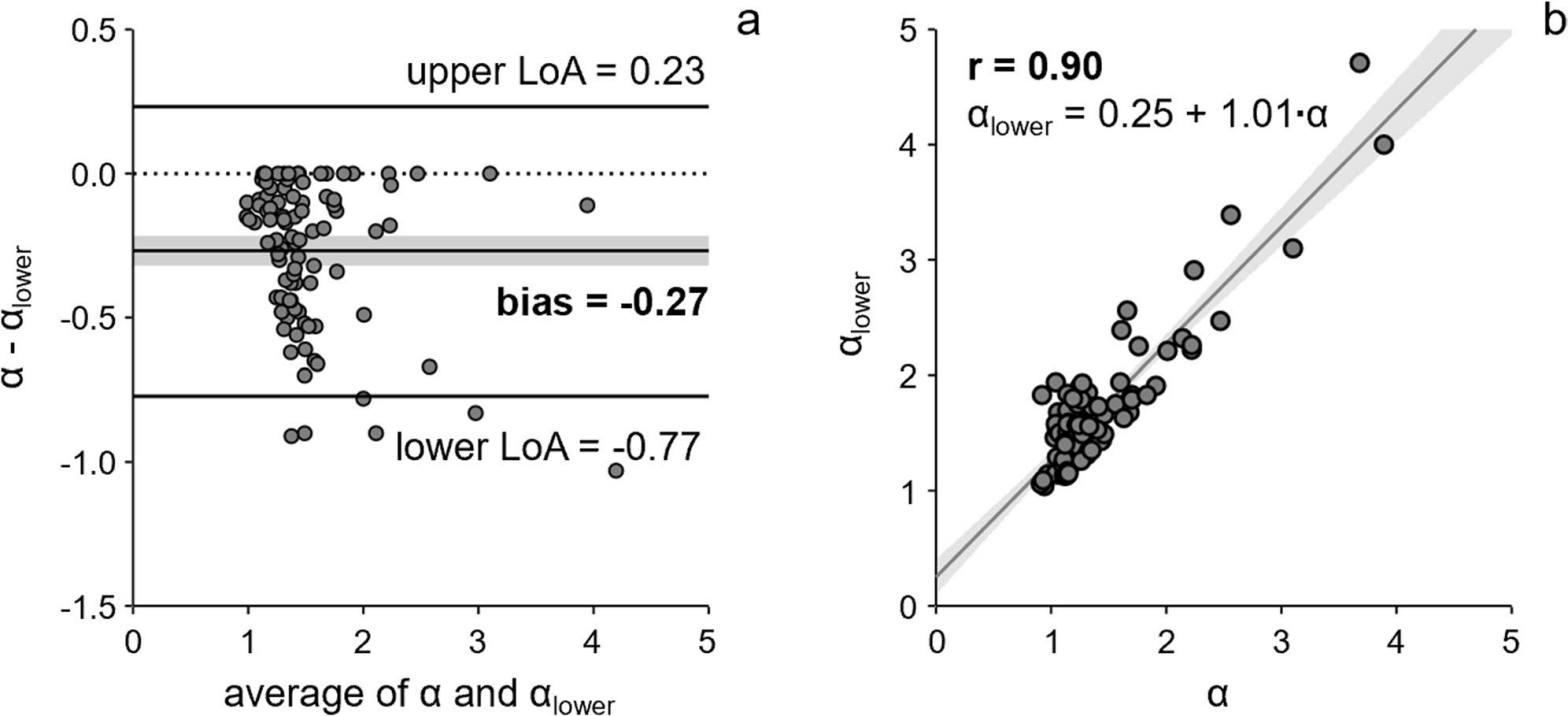
Bland-Altman plot (**a**) and scatter plot with linear regression analysis (**b**) of the LA acceleration factors derived from all pulmonary veins (*α*) and from only the lower pulmonary veins (*α*_lower_). Gray bar indicates the 95% confidence interval of the bias in the case of the Bland-Altman plot and the 95% confidence limits of the regression line in the case of the scatter plot. LoA, limit of agreement; *r*, correlation coefficient

Means of maximal (*v*_*S*_ + *v*_*D*_) / 2 were smaller when evaluated from the lower pulmonary veins, both in total and for the groups of diastolic (dys)function (*p* = < 0.001–0.045) (Table 2). Consequently, a significant bias of −0.27 (*p* < 0.001) between *α* and *α*_lower_ was observed. The correlation between *α* and *α*_lower_ was, however, very high (*r* = 0.90, *p* < 0.001) (Fig. 5).

The AUC for the detection of advanced diastolic dysfunction from *α*_lower_ was 0.961 (0.899–0.990) and did not differ from the corresponding AUC derived from *α*. With the cut-off value *α*_lower_ ≥ 1.77, sensitivity and specificity for the prediction of advanced diastolic dysfunction were 90% (70–99%) and 89% (80–95%), respectively.

### Interobserver reliability

While the interobserver reliability for the determination of peak velocities ranged from good to excellent, the interobserver reliability of *α* as well as *α*_lower_ was excellent (Table 3).

**Table 3:** Interobserver reliability for MR 4D flow–determined LA peak inflow and outflow velocities and left atrial acceleration factor determined from all or just the lower pulmonary veins. Intra-class correlation coefficients (ICC) are given together with their 95% confidence intervals. *SD*_*w*_, within-subject standard deviation; *v*_*E*_, early diastolic left atrial peak outflow velocity; *v*_*S*_, systolic left atrial peak inflow velocity; *v*_*D*_, early diastolic left atrial peak inflow velocity; *α*, left atrial acceleration factor from all pulmonary veins; *α*_*lower*_, left atrial acceleration factor derived from only the lower pulmonary veins

Parameter	SD_w_	ICC
All pulmonary veins		
*v*_*E*_ (cm/s)	3.4	0.97 [0.92–0.99]
Maximum *v*_*S*_ (cm/s)	3.1	0.95 [0.88–0.98]
Maximum *v*_*D*_ (cm/s)	3.5	0.86 [0.68–0.94]
Maximum (*v*_*S*_ + *v*_*D*_) / 2 cm/s	3.1	0.90 [0.77–0.96]
*α*	0.14	0.96 [0.91–0.99]
Lower pulmonary veins		
Maximum (*v*_*S*_ + *v*_*D*_) / 2 cm/s	3.4	0.83 [0.61–0.93]
*α*_lower_	0.20	0.93 [0.84–0.97]

### LA acceleration factor from echocardiographic measurements *α*_echo_

Means of *E*, *S*, *D*, and (*S* + *D*) / 2 as well as corresponding LA acceleration factor *α*_echo_ for the total study population and for the groups of patients with diastolic dysfunction are summarized in Table 4. Due to inadequate visualization of the pulmonary veins in the apical 4-chamber view, *S* and *D* measurements could not be performed in 5 subjects; among the remaining 89 subjects, the right lower pulmonary vein was evaluated in 81 subjects (91%) and the right upper pulmonary vein in 8 subjects (9%). While low correlations were observed between *v*_*E*_ and *E* (*r* = 0.49, *p* < 0.001), the correlation between maximal (*v*_*S*_ + *v*_*D*_) / 2 and (*S* + *D*) / 2 was negligible (*r* = 0.25, *p* = 0.017).

**Table 4:** Echocardiographically determined early diastolic transmitral peak velocity and pulmonary venous peak velocities, as well as LA acceleration factor derived from these measurements. *E*, early diastolic transmitral peak velocity; *S*, systolic pulmonary venous peak velocity; *D*, early diastolic pulmonary venous peak velocity; *α*_*echo*_, LA acceleration factor derived from echocardiographic measurements

Parameter	Total (*n *= 86)	Grade 0 (*n *= 51)	Indet. (*n *= 9)	Grade I (*n *= 13)	Grade II (*n *= 9)	Grade III (*n *= 7)	*p*
*E* (cm/s)	82.0±20.7	77.6±15.8^3^	79.0±15.0^3^	74.4±13.0^3^	103.8±31.5^3^	110.9±18.8^0,i,1^	< 0.001
*S* (cm/s)	55.8±14.8	57.5±11.9	55.3±5.0	56.0±17.5	61.4±21.8	36.3±15.7	0.005
*D* (cm/s)	48.7±13.1	43.7±8.0	46.1±8.7	53.3±11.2	61.0±16.0	63.6±23.0	< 0.001
(*S* + *D*) / 2 (cm/s)	51.2±12.3	50.6±8.4	50.7±6.4	54.7±12.5	61.2±15.2	49.9±19.0	0.719
*α*_echo_	1.64±0.51	1.58±0.42	1.56±0.28	1.44±0.49	1.74±0.54	2.44±0.75	< 0.001

**Fig. 6:**
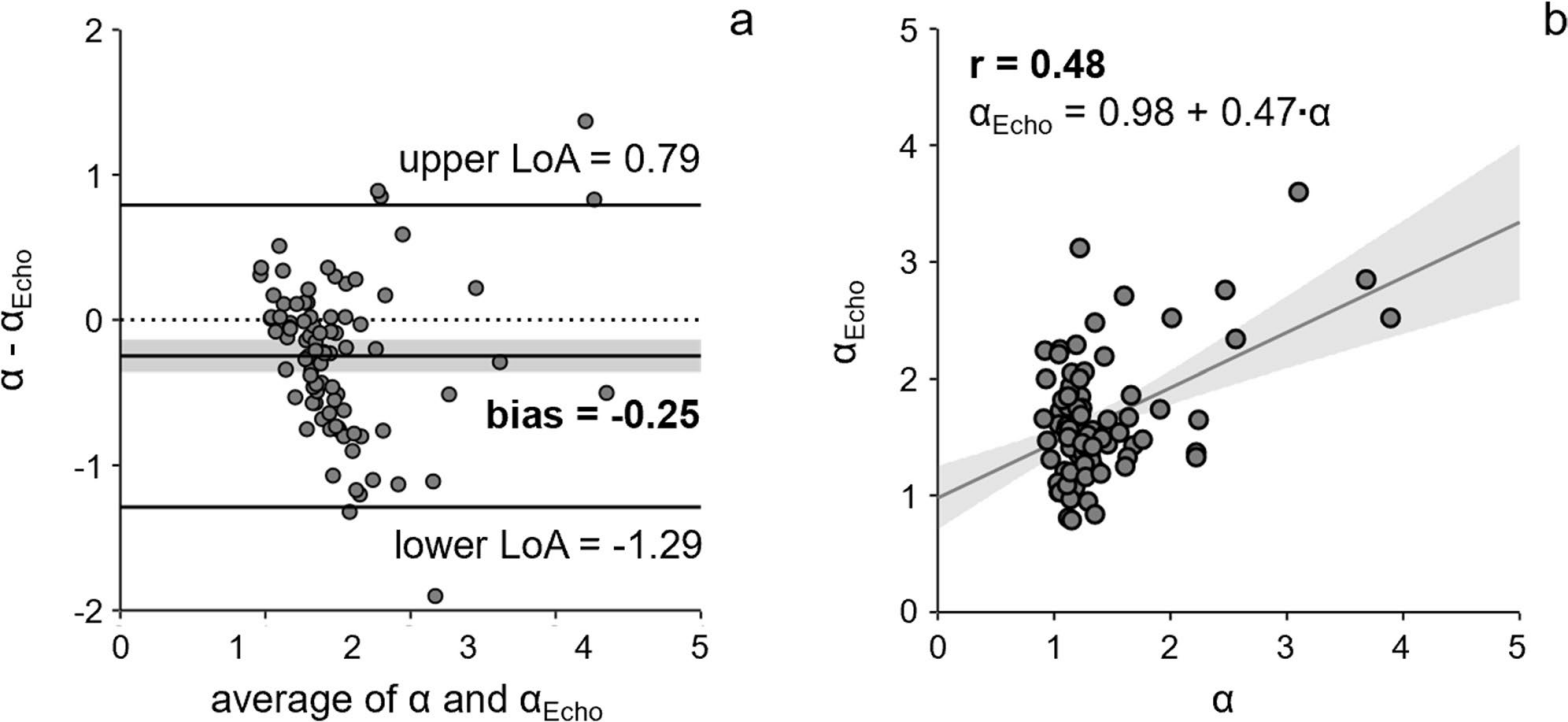
Bland-Altman plot (**a**) and scatter plot with linear regression analysis (**b**) of the LA acceleration factors derived from 4D flow (*α*) and from echocardiography (*α*_echo_). Gray bar indicates the 95% confidence interval of the bias in the case of Bland-Altman plot and the 95% confidence limits of the regression line in the case of the scatter plot. LoA, limit of agreement; *r*, correlation coefficient

*α* and *α*_echo_ showed a significant bias (−0.25, *p* < 0.001), large standard deviation of differences (SD = 0.53), and a low correlation (*r* = 0.48, *p* < 0.001) (Fig. 6). The AUC for the detection of advanced diastolic dysfunction from *α*_echo_ was 0.692 (0.535–0.848).

## Discussion

Our study yielded three main findings: The 4D flow–derived LA acceleration factor *α* (1) allows differentiation of grade II and III diastolic dysfunction from normal diastolic function as well as from non-advanced grades of diastolic dysfunction, (2) shows a very high correlation but a bias to the LA acceleration factor *α*_lower_ determined from the lower pulmonary veins, and (3) is only weakly correlated with *α*_echo_ derived from echocardiographic measurements.

The 4D flow–derived LA acceleration factor *α* was initially introduced as a non-invasive means of estimating PAWP in patients at risk for or with pulmonary hypertension [5]; the present study confirms the association between *α* and PAWP in a heterogeneous cohort of patients by showing that *α* allows differentiation between normal, indeterminate, or grade I diastolic dysfunction and advanced diastolic dysfunction, in which LV filling pressure can be assumed to be increased. *α* showed significant but only low to moderate correlations with echocardiographic *E*, *E*/*A*, *E*/*e*ʹ, and LAVI. Comparable correlations have previously been found between echocardiography and invasively measured PAWP [11, 12], further supporting an association between *α* and PAWP in the current study population, which included subjects with normal LV EF as well as patients with hypertrophic cardiomyopathy, restrictive cardiomyopathy, or aortic stenosis.

Evidence of advanced diastolic dysfunction—or increased LV filling pressures—is not only an important aspect in the diagnosis of heart failure (especially when LV EF is preserved), but also identifies patients at increased risk for cardiovascular events and mortality in a wide variety of conditions [13–15], e.g., in patients on hemodialysis [16, 17], pre-dialysis chronic kidney disease [18], in patients with reduced and normal LV EF [19, 20], patients with ischemic heart failure [21], after cardiac surgery [22], or after transcatheter aortic valve replacement [23, 24].

The established method for non-invasive detection of elevated LV filling pressures is a complete grading of LV diastolic dysfunction using a complex multi-parametric echocardiographic algorithm with thresholds for *E*, *E*/*A*, *E*/*e*ʹ, *e*ʹ, TR, and LAVI [2]; cardiac MR imaging–based assessment mimicking the echocardiographic diagnostic flowchart is possible [6, 25, 26] but currently not established. The MR 4D flow–derived parameter *α* provides a conceptually simple and reliable single-parameter solution for this task, which other known parameters (from echocardiography or from MR) cannot fulfill with comparable accuracy: The diagnostic accuracy of the most commonly used diastolic parameter *E*/*e*ʹ is limited in subjects with normal LV EF [27, 28] as well as in patients with pulmonary hypertension [29, 30], patients with hypertrophic cardiomyopathy [31], and in acutely decompensated patients with reduced EF [32]. The association between enlarged LAVI and elevated LV filling pressure is impaired in—among others—athletes, patients with mitral valve disease, patients with atrial fibrillation [2, 33], and patients with persistent LA dilatation after heart failure therapy [34]. While reduced LA strains have been shown to better predict elevated LV filling pressures compared with other single diastolic parameters [33, 35–37], the diagnostic accuracy of LA strains is nevertheless inferior to that of *α*.

In the model proposed in [5] for non-invasive estimation of PAWP, *α*_lower_ was calculated from the maximum pulmonary venous inflow velocities of the lower left or right pulmonary veins. In the present study, *α* was derived from the maximum pulmonary venous inflow velocities among all pulmonary veins. Interestingly, in the majority of subjects, the highest (*v*_*S*_ + *v*_*D*_) / 2 value was measured in the upper left or right pulmonary vein. Due to the very high correlation between *α* and *α*_lower_, no significant difference was observed for the discrimination of advanced diastolic dysfunction from *α* and *α*_lower_, suggesting that the diagnosis of advanced diastolic dysfunction could in principle be determined in a shorter examination time from a 4D flow stack that does not cover the entire LA volume. It should, however, be noted that due to the observed bias between *α* and *α*_lower_, calculation of PAWP from the formula PAWP = −6.2 + 10.1·*α*_lower_ given in [5] underestimates PAWP by approximately 3 mmHg when used for *α* (e.g., a cut-off of *α* = 1.58 for advanced diastolic dysfunction translates to a PAWP of 13 rather than 10 mmHg, matching well with pressures observed by Andersen et al [12] for different grades of diastolic dysfunction).

The small but significant higher *α* in the grade I diastolic dysfunction group compared with the normal diastolic function group and the significant AUCs for differentiation between normal and grade I diastolic (dys)function within the non-advanced diastolic dysfunction group might indicate a slightly higher PAWP in grade I diastolic dysfunction that is still within in the normal range. The *α* difference of 0.14 between normal and grade I diastolic (dys)function would translate into a calculated PAWP difference of approximately 1.5 mmHg, which was similarly invasively determined in [38].

The echocardiographic analog *α*_echo_ of *α*, derived via the recommended procedure to measure peak pulmonary venous velocities *S* and *D* as well as early diastolic transmitral peak velocity *E*, neither correlated well with *α* nor allowed accurate detection of advanced diastolic dysfunction. Apart from the facts that (*S* + *D*) / 2 was only derived from one pulmonary vein and that pulmonary venous flow is not always assessable with optimal quality by transthoracic echocardiography [39, 40], these findings can be attributed largely to the difference in anatomic localization of LA inflow and outflow velocity measurements on echocardiography as opposed to MR imaging. As shown by measurements of peak velocities within 4D flow datasets, correlation of PAWP and LA acceleration factor dramatically decreases if velocities are not derived at the atrioventricular junction and the orifice of the pulmonary veins but at the mitral valve tips and 1 cm into the pulmonary vein [5].

The study did not aim to optimize time of 4D flow acquisition or the analysis time. The multislice 4D flow protocol was chosen to optimize the “anatomical” contrast in native 4D flow acquisitions [41]. Apart from the aforementioned reduction of the stack volume, acquisition time could be further reduced by using effective navigator gating and/or compressed sensing techniques [42, 43]. This could allow acquisition of 4D flow after contrast application and before late-enhancement imaging. The 4D flow evaluation time included the measurement of systolic and early diastolic peak velocities in all pulmonary veins as well as their anatomical categorization. Determination of the fastest flow or at least two candidates for the fastest flow can be typically performed visually, and their categorization is not required for calculation of *α*. Thus, even without any automation, a potential halving of the evaluation time would be achievable.

We acknowledge the following limitations of our study: It was a single-center proof-of-principle study that investigated the potential of *α* to predict advanced LV diastolic dysfunction. Invasive hemodynamic measurements were not available for the study cohort. The time difference between the cardiac MR imaging study and the echocardiography was kept short but changes in filling pressures may have occurred. Moreover, patients with irregular heart rhythm, patients with significant mitral stenosis, or patients with congenital heart disease in whom echocardiographic assessment of LV diastolic function is limited [2] were not included in the study. Therefore, the results are not applicable to such subjects. Finally, it should be mentioned that *α* was evaluated from native and post-contrast 4D flow datasets. However, the application of contrast agent should not directly affect velocities measured by MR phase-contrast imaging [44].

In conclusion, the 4D flow–derived LA acceleration factor *α*, on its own, allows grade II and grade III diastolic dysfunction to be distinguished from non-advanced grades as well as from each other. Reducing the 4D flow dataset to cover the lower pulmonary veins when calculating *α* is associated with a bias of this parameter but appears to cause only a non-significant decrease in predictive accuracy. The LA acceleration factor derived from standard transmitral and pulmonary venous echocardiographic measurements, on the other hand, cannot be used to distinguish advanced diastolic dysfunction.

## Abbreviations

4D flow: Four-dimensional (time-resolved, three-directional) phase-contrast imaging
*α*: Left atrial acceleration factor
*α*_echo_: Left atrial acceleration factor assessed from echocardiography
*α*_lower_: Left atrial acceleration factor assessed from lower pulmonary veins
*A*: Late diastolic transmitral peak velocity
AUC: Area under the receiver operating characteristic curve
*D*: Early diastolic pulmonary venous peak velocity
*E*: Early diastolic transmitral peak velocity
*e*ʹ: Early diastolic mitral valve tissue peak velocity
EF: Ejection fraction
ICC: Intra-class correlation coefficients
LA: Left atrium
LAVI: Left atrial volume index
LDL: Low-density lipoprotein
LV: Left ventricle
MR: Magnetic resonance
NTproBNP: N-terminal prohormone of brain natriuretic peptide
PAWP: Mean pulmonary arterial wedge pressure
ROC: Receiver operating characteristic curve
*S*: Systolic pulmonary venous peak velocity
SD_w_: Within-subject standard deviation
sPAP: Systolic pulmonary arterial pressure
TR: Peak tricuspid regurgitation velocity
*v*_*D*_: Early diastolic left atrial peak inflow velocity
*v*_*E*_: Early diastolic left atrial peak outflow velocity
*v*_*S*_: Systolic left atrial peak inflow velocity

## Declarations

### Guarantor

The scientific guarantor of this publication is Ursula Reiter.

### Statistics and biometry

One of the authors (Gert Reiter) has significant statistical expertise. No complex statistical methods were necessary for this paper.

### Study subjects or cohorts overlap

• 4D flow data of 10 subjects of the study population were included in the definition of an algorithm for automated mitral valve vortex ring extraction from 4D flow MRI (Kräuter C et al, MRM 84 (2020). https://doi.org/10.1002/mrm.28361).

• The impact of breath-holding on cine real-time volumetric function parameters was analyzed previously from 56 subjects of this study population (Reiter C et al, Eur J Rad 141 (2021). https://doi.org/10.1016/j.ejrad.2021.109756).

• Volumetric function parameters of 60 subjects were analyzed from native multislice 4D flow data and compared to cine real-time imaging (Reiter C et al, Eur Radiol 2023; https://doi.org/10.1007/s00330-023-10017-3).

• 4D flow evaluation strategies for assessment of transmitral and myocardial velocities were analyzed in 60 subjects with echocardiography as reference; a manuscript is currently under review in the *European Journal of Radiology*.

### Methodology

• prospective study

• diagnostic study

• performed at one institution
